# Collagen I in the Hip Capsule Plays a Role in Postoperative Clinical Function in Patients With Developmental Dysplasia of the Hip

**DOI:** 10.3389/fped.2022.918660

**Published:** 2022-05-11

**Authors:** Sicheng Zhang, Jun Song, Qingjie Wu, Jihong Fang, Bo Ning

**Affiliations:** ^1^Department of Pediatric Orthopaedic, Anhui Provincial Children’s Hospital, Hefei, China; ^2^Children’s Hospital, Fudan University, Shanghai, China; ^3^Anhui Provincial Children’s Hospital, Hefei, China

**Keywords:** developmental dysplasia of the hip (DDH), collagen I, hip capsule, joint function, surgery

## Abstract

The aims of the present study is to evaluate the roles of collagen I and III in the hip capsule in the postoperative clinical function of patients with developmental dysplasia of the hip (DDH). Hip capsules from 155 hips of 120 patients were collected during surgery. The patients were divided into three groups according to age: I: 2–3.5 years; II: 3.5–5 years; and III: 5–6 years. Patient clinical function and radiographic outcomes were evaluated with the McKay scores and Severin classification. The expression of collagen I and III was detected through immunohistochemistry and quantitative reverse transcription polymerase chain reaction (RT-PCR) and analyzed according to age, sex, degree of dislocation and McKay classification. All patients received open reduction and pelvic osteotomy and/or femoral shortening osteotomy and achieved good results on the basis of postoperative X-ray imaging. The average follow-up time was 3.4 years (range 2–4.3 years). There were no changes in the expression of collagen III in the different groups. The expression of collagen I according to age and sex was not significantly different. Lower expression of collagen I was observed in DDH patients with a higher degree of dislocation according to the Tonnis grade. The highest expression of collagen I was detected in the group with poor clinical function according to the McKay classification. Collagen I is correlated with the degree of dislocation and is a risk factor for poor clinical function in DDH patients. Collagen I is correlated with the degree of hip dislocation and poor clinical function in DDH patients.

## Introduction

Developmental dysplasia of the hip (DDH) is one of the most common deformities in children (1–3). Pathological changes in the acetabulum, proximal femur, and joint capsule have been observed (4, 5). During DDH treatment, the bone deformities are corrected, and the capsule is partly removed and tightened (5, 6). Bone changes can be detected by X-ray imaging; however, the molecular changes in the hip joint capsule are still unknown.

The joint capsule is mainly composed of collagen and a small number of fibroblasts (7, 8). The major collagens in the capsule are types I and III, with the former making up 83% of the collagen present (9). Collagen type I is located in tissues that require high levels of mechanical strength, and it is abundant at sites where new fibrosis and connective tissue proliferation occur (10, 11). Collagen type III is involved in the development and differentiation of mesenchymal tissue, and it is present in large quantities in tissues that require high levels of mechanical compliance (11, 12). Determination of the expression of collagen types I and III is valuable for assessing whether changes in the joint capsule indicate the development of arthrochalasis (13). Children with DDH generally experience different degrees of joint laxity, with a lower incidence in whites and higher incidence in females (14, 15). However, the changes in collagen types I and III in DDH capsules and their relation to clinical manifestations remain unknown.

To investigate the changes in collagen types I and III in DDH capsules, we retained DDH capsule specimens during surgery. The expression of collagen I and III in the specimens was detected by immunohistochemistry and quantitative (q)RT-PCR. We also investigated the relationship between the expression of collagen I and III and the degree of hip dislocation and function. To the best of our knowledge, this is the first study to detect the relationship between DDH capsules and clinical manifestations.

## Materials and Methods

### Patients and Materials

In this retrospective study, we enrolled 128 children > 2 years of age with DDH diagnosed between March 2014 and June 2016. We excluded eight patients with poor results during follow-up X-ray imaging which may interfere the evaluation of clinical function and affect our results. In the remaining 120 patients (155 hips), the mean age was 4.6 years (range 2–6 years); 95 patients with 125 hips were female, and 25 patients with 30 hips were male; 85 cases presented with unilateral DDH and 35 with bilateral; 98 patients were from the left side and 57 from the right side. The patients were divided into three groups according to age: I: 2–3.5 years; II: 3.5–5 years; and III: 5–6 years. The degree of dislocation was evaluated with Tonnis’ classification (16, 17) before surgery. The study was approved by the Ethical Committee of Anhui Provincial Children’s Hospital (20130022). Verbal consent was obtained from the legal guardian of the children, and signed informed consent on the surgical consent form.

### Clinical Data Collection

All the patients received open reduction and pelvic osteotomy (Salter or Pemberton) and/or femoral shortening osteotomy from two senior orthopedic surgeons. The hip capsules were collected for immunohistochemistry and qRT-PCR. The patients received half hip cast immobilization for 6 weeks followed by 2 weeks of weight-free physical therapy, and then walked freely. The average follow-up time was 3.4 years (range 2–4.3 years). The clinical function and radiographic outcomes at the lasted follow-up were evaluated with McKay’s scoring system and the Severin classification, respectively (18, 19).

### Immunohistochemistry

The capsules isolated from DDH hips were fixed in 4% paraformaldehyde and embedded in paraffin. Five representative sections (4 μm) from each joint capsule obtained from various depths were mounted on slides. The sections were deparaffinized in xylene, rehydrated, and washed three times with phosphate buffered saline for 5 min each at room temperature. Endogenous peroxidase activity was blocked by 3% hydrogen peroxide for 10 min. The slides were subjected to antigen retrieval by microwave irradiation in 10 mM sodium citrate (pH 6.0) for 10 min. Sections were incubated overnight at 4°C with either monoclonal rabbit anti-human collagen I or rabbit anti-human collagen III antibody (Abcam, Cambridge, MA, United States) at a dilution of 1:200. In the negative control reaction, the primary antibody was omitted. Thereafter, sections were analyzed with an EnvisionTM Detection Kit (Dako, Glostrup, Denmark), treated with 3,3’-diaminobenzidine and counterstained with Mayer’s haematoxylin. Slides were visualized under a microscope. The integrated optical density was measured in 5 randomly chosen regions of each section using Image Pro Plus.

### Ribonucleic Acid Extraction and Quantitative Reverse Transcription-Polymerase Chain Reaction

Total RNA was extracted from the capsules using TRIzol reagent (Invitrogen Life Technologies, Paisley, United Kingdom). The purity and amount of RNA were determined by measuring the OD_260/280_ ratio. Preservation of 28S and 18S rRNA was used to assess RNA integrity.

Reverse transcription of 1 μg RNA to complementary deoxyribonucleic acid (cDNA) was performed using a ReverTra Ace qPCR RT kit (TOYOBO, Osaka, Japan). The yields were quantified spectrophotometrically. qRT-PCR was performed using 5 μl cDNA (100 ng), 2 μl each primer (10 μM), 25 μl SYBR Green Realtime PCR Master Mix (TOYOBO), and 16 μl water in a total volume of 50 μl. The target genes were Collagen I and Collagen III, whose expressions were normalized to that of the housekeeping gene β*-actin*. The primers are shown in Table 1. qRT-PCR was performed using an ABI PRISM 3730HT Sequence Detection System (Vernon, CA, United States), which was programmed to an initial step of 10 min at 95°C for polymerase activity, followed by 40 cycles of 15 s denaturation at 95°C, 15 s annealing at 60°C, and 45 s extension at 72°C. The absence of non-specific Polymerase Chain Reaction (PCR) products was verified using melting curve and electrophoresis analyses. Reactions were performed in triplicate, and the average values were used. The relative quantification of target genes was determined using the ΔΔCT method. The results are expressed as the fold change in expression of the target gene relative to that of the housekeeping gene.

**TABLE 1:** Primers used for amplification of target genes and β-actin.

	F-Sequence (5′-3′)	R-Sequence (5′-3′)
Collagen I	GGGAACATCCTCCTTCAACAG	GGAGCTGGCTACTTCTCGC
Collagen III	CAGATCACGTCATCGCACAAC	GAGGGCCAAGACGAAGACATC
β-actin	CCTCGCCTTTGCCGATCC	GGATCTTCATGAGGTAGTCAGTC

### Statistical Analysis

Descriptive statistical analysis was performed using mean values and standard deviations. Data were analyzed with the statistical software SPSS 16.0, and two-tailed Student’s *t* tests were used. *P* < 0.05 was considered statistically significant.

## Results

### Clinical Data and Follow-Up Results

One hundred and twenty patients (150 hips) were followed up and evaluated on the basis of X-ray imaging and clinical function. Based on Severin radiographic classification (19), the radiographic results were excellent or good. Clinical data and McKay’s classification (18) results are shown in Table 2. The patients were divided into three groups according to Tonnis’ classification (16, 17) and their preoperative X-ray films: 24 hips were Tonnis I and II, 48 were Tonnis III, and 83 were Tonnis IV (Table 2).

**TABLE 2:** Clinical characteristics of developmental dysplasia of the hip (DDH) patients.

Characteristics	Tonnis I/II	Tonnis III	Tonnis IV	Total
**Gender**				
Male	8	11	11	30
Female	16	37	72	125
**Age (years)**				
2–3.5	7	19	24	50
3.5–5	8	17	29	54
5.5–6	9	12	30	51
**DDH side**				
Left	12	23	28	63
Right	6	8	8	22
Bilateral	6	17	47	70
Total	24	48	83	155

### Expression of Collagen I and III in Different Basic Clinical Groups

Immunohistochemistry and qRT-PCR showed the same trends in variation in the different groups. There was no significant difference in the expression of collagen I and III according to age group, sex, left and right side, or unilateral and bilateral DDH (*P* > 0.05).

### Expression of Collagen I and III According to Degree of Dislocation

**FIGURE 1:**
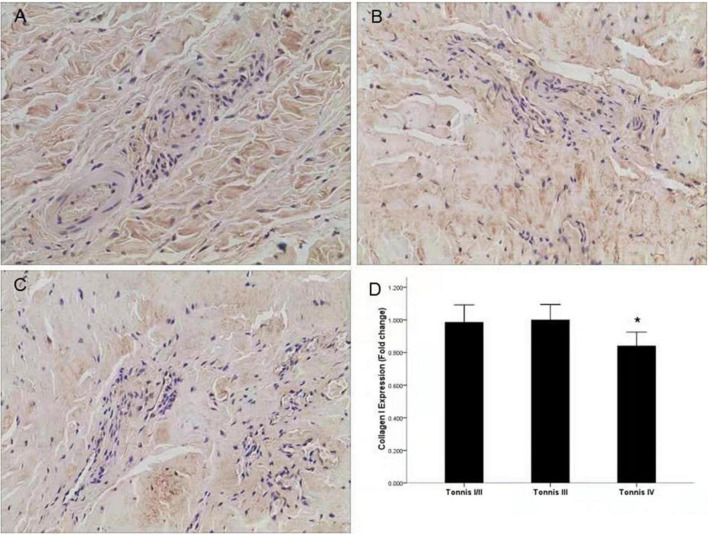
Expression of collagen I in different groups according to the Tonnis classification. **(A)** Representative image of a Tonnis II grade DDH capsule. **(B)** Representative image of a Tonnis III grade DDH capsule. **(C)** Representative image of a Tonnis IV grade DDH capsule, and lower expression of collagen I was observed. **(D)** Quantification of collagen I immunohistochemistry results by measurement of the IOD in different groups. *P < 0.05; Magnification: 10 × 40.

**FIGURE 2:**
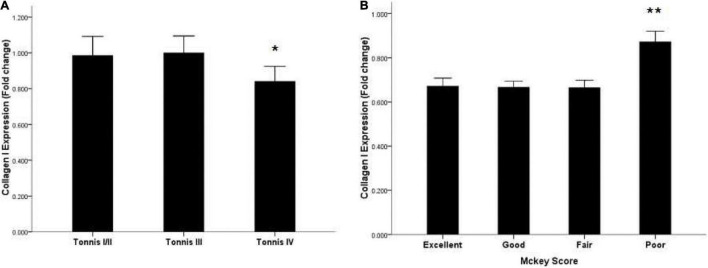
Expression of collagen I mRNA in the capsules of DDH patients according to the Tonnis classification and McKey score. **(A)** Lower expression of collagen I mRNA in Tonnis IV grade patients. **(B)** Highest expression of collagen I mRNA in poor-group patients according to McKey score. *P < 0.05; **P < 0.001.

To investigate the relationship between collagen I and III and the degree of dislocation, we divided the patients into three groups according to the Tonnis classification (16, 17). There were only three hips in Tonnis class I, so Tonnis classes I and II were analyzed as a single group. For collagen III, there were no significant differences among the three classification groups. However, significantly lower expression of collagen I was observed in the Tonnis IV group through immunohistochemistry (Figures 1A–D) and qRT-PCR compared to the other two groups (*P* < 0.05) (Figure 2A).

### Expression of Collagen I and III According to McKay’s Classification

**FIGURE 3:**
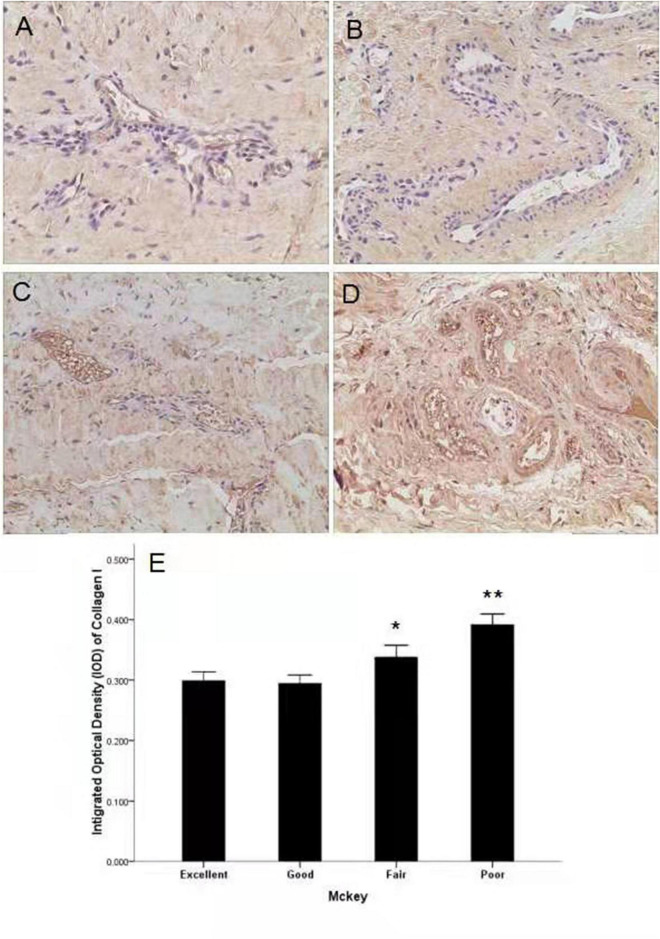
Expression of collagen I in different groups according to the McKey score. **(A)** Representative image of an excellent-group DDH capsule. **(B)** Representative image of a good-group DDH capsule. **(C)** Representative image of a fair-group DDH capsule. **(D)** Representative image of a poor-group DDH capsule and higher expression of collagen I was observed. **(E)** Quantification of collagen I immunohistochemistry results by measurement of the IOD in different groups. *P < 0.05; **P < 0.001; Magnification: 10 × 40.

There was no significant difference in the expression of collagen III as measured by immunohistochemistry and qRT-PCR. However, significantly higher expression of collagen I was observed in patients with poorer joint function compared to the other two groups (*P* < 0.001) (Figures 2B, 3A–E).

## Discussion

Development dysplasia of the hip is one of the most common lower limb deformities, about 12% (1, 2, 20). It is characterized by multiple pathological changes, including in the acetabulum, proximal femur, and soft tissue around the hip (4, 21). Although the DDH capsules are tightened during surgery (4, 5, 22, 23), some patients still have poor clinical function (24). In the present study, all patients underwent similar surgery and physical therapy performed by two senior pediatric orthopedists surgeons and the same physical therapist, respectively. Some patients continued to have poor clinical function of the hip may be result from operation, age, etc., but the confirmed causes are still unclear (22–24). The capsule factors may be of concern. The main components of capsular collagen I and III were detected and analyzed among the different groups according to different factors.

The major types of collagen in the hip joint capsule are I and III (9, 25). Three variations in the *COL1A1* gene promoter have been reported in patients with DDH, and a higher rate of total variation in the *COL1A1* gene contributes to DDH (26). Skirving et al. (27) reported that the ratio of collagen I and III changes in the joint capsule in children with DDH compared with normal children. Hagiwara et al. (28) fixed the knees of rats and detected the expression of collagen I and III at different stages. They found no changes in the expression of collagen I and III or in acquired ankylosis. These studies show that collagen I is important for resistance to tension in the joint capsule. The changes in collagen may result in differing degrees of joint laxity in DDH.

Age and sex are factors that influence the clinical function of the hip joint (22, 29). Male patients often have poorer function than female patients (4, 30). One study showed that sex is an independent factor with a smaller contribution than age to passive stiffness of the hip capsule ligaments (31). In the present study, all of the patients were at a good age for surgery. There were no significant differences among the patients by age or sex. Our patients were all under 6 years of age, and their clinical function was good to excellent for their age. Collagen I and III did not change with age. Our results suggest that collagen I and III in the hip joint capsule may not be correlated with joint function among patients of different ages and sexes.

The degree of joint dislocation is an important risk factor for a poor prognosis of DDH (4, 30), and many studies have shown that a high degree of dislocation results in poor clinical outcomes (19, 30, 32). However, patients under 6 years of age with a high degree of hip dislocation have been found to achieve good results after a one-stage operation with pelvic osteotomy, femur shortening, and capsulorrhaphy (4, 30). Clinical outcomes can be improved by the implementation of surgical techniques in some patients with a high degree of hip dislocation (4, 33). However, there are still some patients who have poor clinical results because of age, gender, congenital diseases, etc (4, 34, 35). We speculated that capsule factors may play a role in clinical outcome. Our patients were divided into three groups according to Tonnis grade (16, 17), and the expression of collagen I and III was analyzed in these different groups. The expression of collagen III did not differ among the groups. However, a lower expression of collagen I was observed in patients with a higher degree of dislocation. The results suggest that collagen I is related to the degree of dislocation in DDH patients. A lower expression of collagen I may lead to joint laxity and a subsequently higher degree of dislocation.

The clinical function of DDH is one of the main evaluation criteria for successful treatment. DDH risk factors include age, degree of dislocation, and AVN (4, 30). Some studies have shown that joint laxity is beneficial for clinical function in DDH (14, 36). Joint laxity may be correlated with the expression of collagen in the hip joint capsule. In the present study, the patients were divided into three groups according to McKay’s classification (18). The expression of collagen III showed no differences among the three classification groups. However, a higher expression of collagen I was observed in the classification group with poor McKay scores. Given that collagen I is associated with DDH and capsule laxity, the higher expression of collagen I in the present study might have resulted in less capsular laxity, which might have led to poor postoperative clinical function of hip joints in patients with DDH. Our results suggest that collagen I plays an important role in the clinical function of DDH.

There were limitations in the present study. First, we need more useful clinical scoring criteria than the McKay classification (18) to evaluate the outcomes of clinical function of the hip. All patients need further and longer follow-up, even up to skeletal maturity, to confirm the ultimate clinical and radiographic outcomes. Further research on the relation between collagen I and the clinical function of postoperative DDH patients will be conducted.

## Conclusion

In summary, differences in the expression of collagen I and III were not observed in DDH patients of different ages and sexes. Collagen I is correlated with the degree of hip dislocation and poor clinical function in DDH patients.

## Data Availability Statement

The raw data supporting the conclusions of this article will be made available by the authors, without undue reservation.

## Ethics Statement

The studies involving human participants were reviewed and approved by Biomedical Ethics Committee of Anhui Provincial Children’s Hospital. Written informed consent to participate in this study was provided by the participants’ legal guardian/next of kin. Written informed consent was obtained from the individual(s), and minor(s)’ legal guardian/next of kin, for the publication of any potentially identifiable images or data included in this article.

## Author Contributions

SZ and JS wrote the manuscript and analyzed the data. SZ and BN performed the experiment. JF and QW analyzed the data and performed the operation. All authors have read and approved the final manuscript.

## Conflict of Interest

The authors declare that the research was conducted in the absence of any commercial or financial relationships that could be construed as a potential conflict of interest.

## Publisher’s Note

All claims expressed in this article are solely those of the authors and do not necessarily represent those of their affiliated organizations, or those of the publisher, the editors and the reviewers. Any product that may be evaluated in this article, or claim that may be made by its manufacturer, is not guaranteed or endorsed by the publisher.
